# Supplementary material for: Experimental witness of quantum jump induced high-order Liouvillian exceptional points
Source: Nat Commun. 2026 Jan 22;17:1923. doi: 10.1038/s41467-026-68705-9 (PMC12923587; doi:10.1038/s41467-026-68705-9)
Supplement: Supplementary file 1 — Supplementary Information [file 41467_2026_68705_MOESM1_ESM.pdf]

Supplementary Materials for "Experimental Witness of Movement of Second- and Third-Order Liouvillian Exceptional Points"

I. ANALYTIC SOLUTION OF THE LINDBLAD EQUATION

The Liouvillian superoperator for our effective two-level ion system is of the form of

$$\begin{aligned}\hat{L}(\alpha) &= \begin{pmatrix} -i\alpha\gamma & -\frac{\Omega}{2} & \frac{\Omega}{2} & 0 \\ -\frac{\Omega}{2} & -i\frac{\gamma}{2}-\Delta & 0 & \frac{\Omega}{2} \\ \frac{\Omega}{2} & 0 & -i\frac{\gamma}{2}+\Delta & -\frac{\Omega}{2} \\ i\alpha\gamma & \frac{\Omega}{2} & -\frac{\Omega}{2} & 0 \end{pmatrix} = \alpha\hat{L}_0 + (1-\alpha)\hat{L}_\phi \\ &= \alpha \begin{pmatrix} -i\gamma & -\frac{\Omega}{2} & \frac{\Omega}{2} & 0 \\ -\frac{\Omega}{2} & -i\frac{\gamma}{2}-\Delta & 0 & \frac{\Omega}{2} \\ \frac{\Omega}{2} & 0 & -i\frac{\gamma}{2}+\Delta & -\frac{\Omega}{2} \\ i\gamma & \frac{\Omega}{2} & -\frac{\Omega}{2} & 0 \end{pmatrix} + (1-\alpha) \begin{pmatrix} 0 & -\frac{\Omega}{2} & \frac{\Omega}{2} & 0 \\ -\frac{\Omega}{2} & -i\frac{\gamma}{2}-\Delta & 0 & \frac{\Omega}{2} \\ \frac{\Omega}{2} & 0 & -i\frac{\gamma}{2}+\Delta & -\frac{\Omega}{2} \\ 0 & \frac{\Omega}{2} & -\frac{\Omega}{2} & 0 \end{pmatrix},\end{aligned} \tag{S1}$$

where $\alpha = \gamma_0/(\gamma_0+\gamma_\phi)$ denotes the ratio of decay rate γ_0 with respect to the total dissipation rate $\gamma = \gamma_0 + \gamma_\phi$, containing the dephasing rate γ_ϕ, Ω represents the Rabi frequency of the effective two-level ion with a detuning Δ. Then, we obtain three non-trival eigenvalues of Liouvillian superoperator as

$$E_{n=1,2,3} = \frac{1}{6}\left(-3i\gamma + iA + e^{i(\frac{1}{2}-\frac{2}{3}n)\pi} Y/\sqrt[3]{-iAX + i\sqrt{A^2X^2-Y^3}} - e^{i(-\frac{1}{2}+\frac{2}{3}n)\pi}\sqrt[3]{-iAX + i\sqrt{A^2X^2-Y^3}}\right) \tag{S2}$$

with

$$\begin{aligned} A &= (1-2\alpha)\gamma \\ X &= (1-2\alpha)^2\gamma^2 + 36\Delta^2 - 18\Omega^2. \\ Y &= (1-2\alpha)^2\gamma^2 - 12(\Delta^2+\Omega^2) \end{aligned} \tag{S3}$$

as well as a trivial solution $E_4 = 0$.

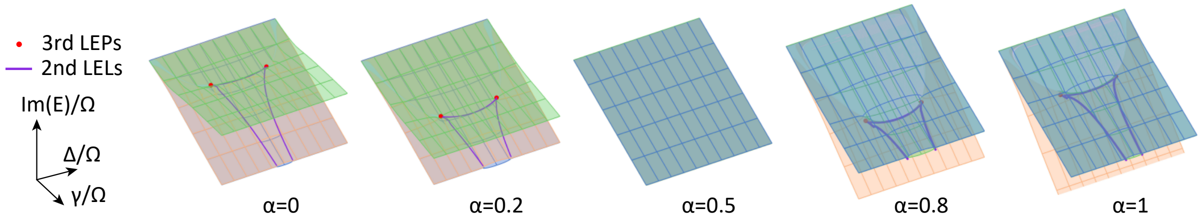

Supplementary Fig. 1. **Imaginary parts of eigenenergies.** (a) Variation of the second-order and third-order Liouvillian exceptional points with respect to α. The colored surfaces are imaginary parts of the complex eigenenergy Riemann surfaces with the orange, blue and green surfaces corresponding, respectively, to the eigenenergies E_1, E_2 and E_3. LEPs: Liouvillian exceptional points; LELs: Liouvillian exceptional lines.

Using Eq. (S2), we plot the imaginary parts of eigenvalues $Im(E)$ for $E = E_{n=1,2,3}$ in Supplementary Fig. 1. These imaginary parts $Im(E)$ form a Riemann surface structure, revealing the second-order Liouvillian exceptional lines in purple and two third-order Liouvillian exceptional points (LEPs) in red.

These degeneracies correspond to equally complex eigenenergies satisfying $A^2X^2 - Y^3 = 0$, governed by

$$\frac{\gamma}{\Omega} = \sqrt{\frac{-8(\frac{\Delta}{\Omega})^4 + 20(\frac{\Delta}{\Omega})^2 + 1 \pm \sqrt{(-8(\frac{\Delta}{\Omega})^2 + 1)^3}}{2(\frac{\Delta}{\Omega})^2}} \Big/ |1 - 2\alpha|. \tag{S4}$$

Therefore, with the increase of α, we observe that 3rd-LEPs move from $\gamma/\Omega = \sqrt{27/2}$ at $\alpha = 0$, to $\gamma/\Omega = 5\sqrt{6}/4$ at $\alpha = 0.2$, then to $\gamma/\Omega = \infty$ at $\alpha = 0.5$, followed by a return to $\gamma/\Omega = 5\sqrt{6}/4$ at $\alpha = 0.8$, and finally to $\gamma/\Omega = \sqrt{27/2}$ for $\alpha = 1$. Such movement results from the non-commutativity of the Liouvillian superoperators for decay $\hat{L}_0$ and dephasing $\hat{L}_\phi$ (i.e., $[\hat{L}_0, \hat{L}_\phi] \neq 0$).

II. MEASUREMENT OF COMPLEX EIGENENERGIES BY TOMOGRAPHY

In our system, the dynamics is governed by $|\Psi(t)\rangle = \sum_{n=1}^{4} |\psi_n\rangle e^{-iE_n t}$ for $n = 1, 2, 3, 4$. Since the imaginary parts $Im(E)$ correspond to dissipation, the final state of our system must evolve to the steady state $|\Psi(t = \infty)\rangle = |\psi_4\rangle$ for $E_4 = 0$ exhibiting no decay. The steady state $|\psi_4\rangle$ is of the form of

$$|\Psi(\tau)\rangle = \left(\frac{\Omega^2}{\alpha\gamma^2 + 4\alpha\Delta^2 + 2\Omega^2}, \frac{2\alpha\Delta\Omega - i\alpha\gamma\Omega}{\alpha\gamma^2 + 4\alpha\Delta^2 + 2\Omega^2}, \frac{2\alpha\Delta\Omega + i\alpha\gamma\Omega}{\alpha\gamma^2 + 4\alpha\Delta^2 + 2\Omega^2}, \frac{\alpha\gamma^2 + 4\alpha\Delta^2 + \Omega^2}{\alpha\gamma^2 + 4\alpha\Delta^2 + 2\Omega^2}\right)^T,$$

which corresponds to a density matrix

$$\rho_4 = \begin{pmatrix} \frac{\Omega^2}{\alpha\gamma^2 + 4\alpha\Delta^2 + 2\Omega^2} & \frac{2\alpha\Delta\Omega - i\alpha\gamma\Omega}{\alpha\gamma^2 + 4\alpha\Delta^2 + 2\Omega^2} \\ \frac{2\alpha\Delta\Omega + i\alpha\gamma\Omega}{\alpha\gamma^2 + 4\alpha\Delta^2 + 2\Omega^2} & \frac{\alpha\gamma^2 + 4\alpha\Delta^2 + \Omega^2}{\alpha\gamma^2 + 4\alpha\Delta^2 + 2\Omega^2} \end{pmatrix}. \tag{S5}$$

Experimentally, we perform 14,000 repeated measurements on steady-state configurations to complete the tomography of the experimental points. With the original dataset of 14,000 data points, we randomly sample 10,000 points per group, fit each group individually, and repeat this process for 120 times to calculate the average values and error bars. Using Eq. (S5), we fit γ and α with 120 groups of tomographic data by setting the constants Ω and Δ. This allows us to calculate the averages γ_{mean} and α_{mean}, with their error bars derived from the uncertainties in γ and α. Similarly, we determine the complex eigenenergies E and their error bars by substituting γ and α into Eq. (S2). We present subset of experimental data for steady-state tomography in Supplementary Fig. 2(b-j) when $\Delta = 0$ and in Supplementary Fig. 3(b-j) when $\Delta = \Omega/\sqrt{8}$. Since in pure dephasing system, the matrix elements of the steady-state tomography for different γ are of the same values, we measure time evolution of the population in $|e\rangle$ in z-direction from 0 to 45 μs in Supplementary Fig. 2(a) when $\Delta = 0$ and in Supplementary Fig. 3(a) when $\Delta = \Omega/\sqrt{8}$.

Next, we measure the eigenvalues working as a function of the decay rate γ while keeping α constant. We show two typical eigenvalue measurements around the second-order Lindblad LEPs for $\Delta/\Omega = 0$ in Supplementary Fig. 4(a,b) and around the third-order Lindblad LEPs for $\Delta/\Omega = 1/\sqrt{8}$ in Supplementary Fig. 4(c,d) LEP with $\alpha \approx 0.1$. Here the degenerate eigenvalues correspond to LEPs. Due to the high sensitivity of LEPs to parameter variations, we set the experimental parameters to approach these points as closely as possible and observe their signatures via eigenvalues.

This method allows us to track the movement of LEPs across parameter spaces by varying values of α in both experiments and simulations. Except for the observed movement of the second-order (third-order) LEPs for $\Delta/\Omega = 0$ ($\Delta/\Omega = 1/\sqrt{8}$) in the main text, we also observe the movement of a third-order LEP

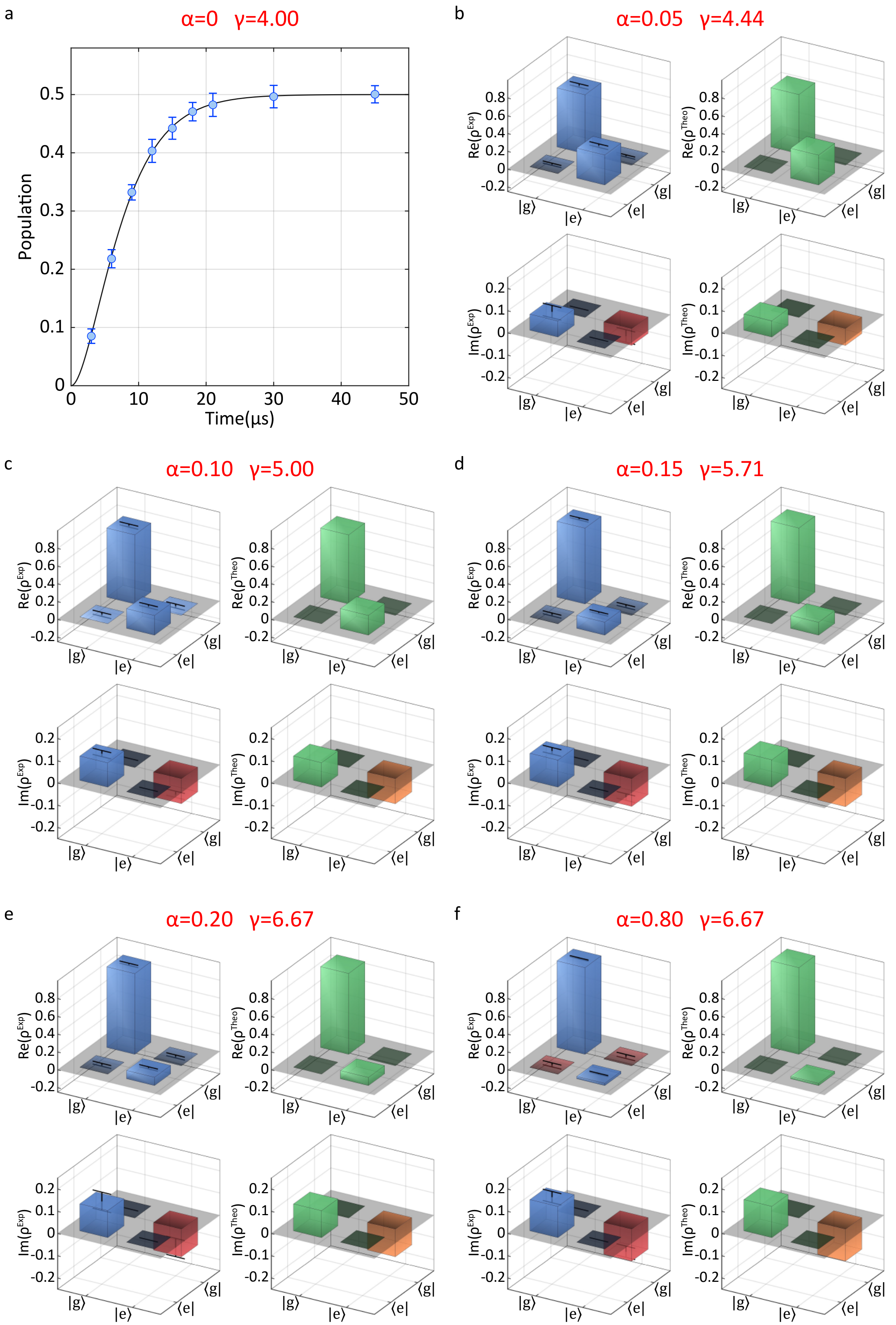
a
α=0 γ=4.00
Population
Time(μs)
b
α=0.05 γ=4.44
c
α=0.10 γ=5.00
d
α=0.15 γ=5.71
e
α=0.20 γ=6.67
f
α=0.80 γ=6.67
$Re(\rho^{Exp})$
$Re(\rho^{Theo})$
$Im(\rho^{Exp})$
$Im(\rho^{Theo})$
|g⟩
|e⟩
⟨g|
⟨e|

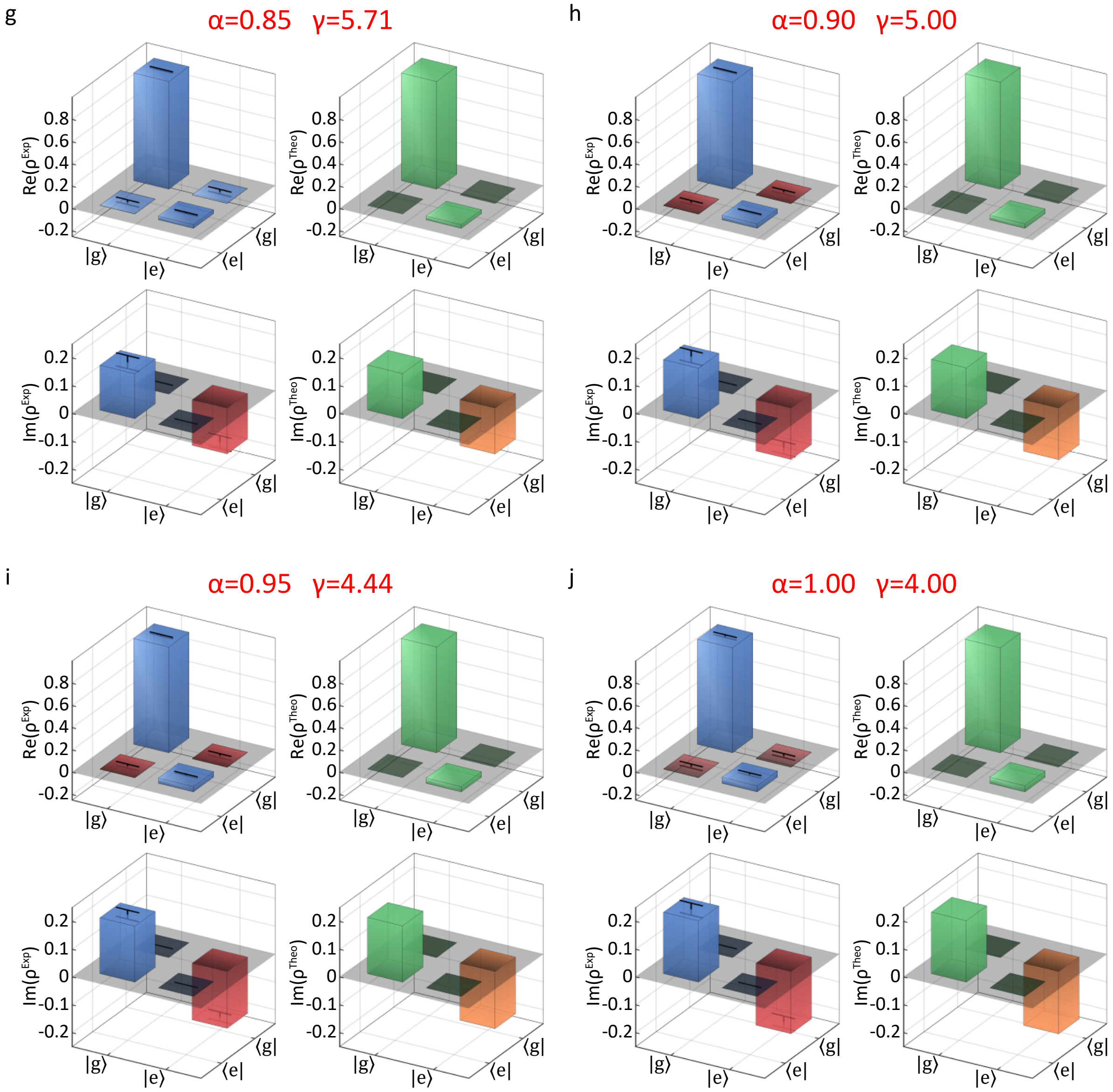

Supplementary Fig. 2. **Time evolution of the population as well as the steady-state tomography when $\Delta = 0$.** (a) Time evolution of the population in $|e\rangle$ in z-direction with error bars. (b-j) The steady-state tomography with different α. The upper panels show the real parts while the lower panels display the imaginary parts. In each panel, the lefthand side are the experimental values with error bars and the righthand side are the theoretical values. Positive values are represented in blue(green) for experimental(theoretical) values, while negative values are shown in red(orange) to facilitate distinction. The error bars are standard deviation representing the statistical errors of 14 000 measurements for each data point.

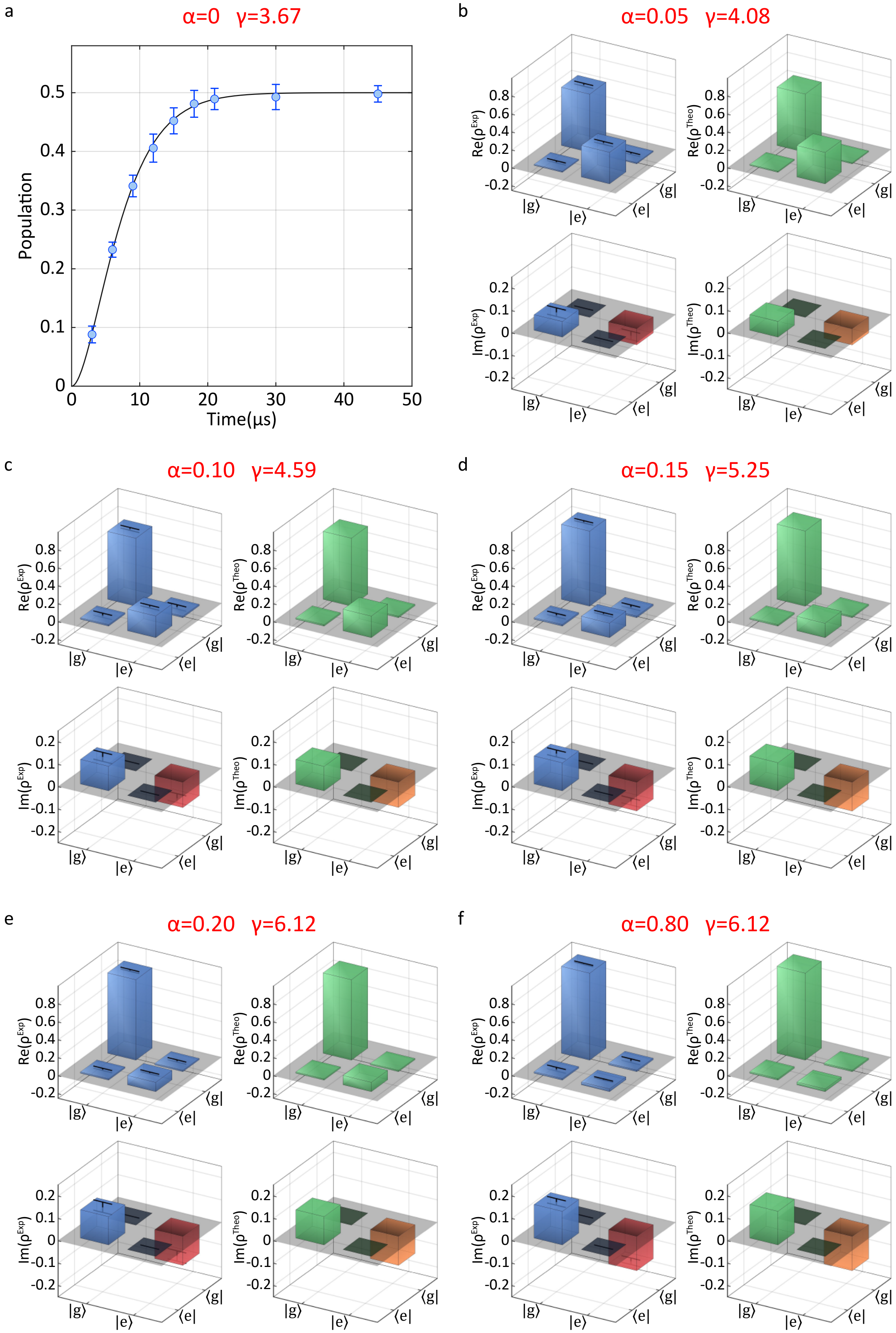

a
α=0 γ=3.67
Population
Time(μs)
b
α=0.05 γ=4.08
c
α=0.10 γ=4.59
d
α=0.15 γ=5.25
e
α=0.20 γ=6.12
f
α=0.80 γ=6.12
Re(ρ^Exp)
Re(ρ^Theo)
Im(ρ^Exp)
Im(ρ^Theo)
|g⟩
|e⟩
⟨g|
⟨e|

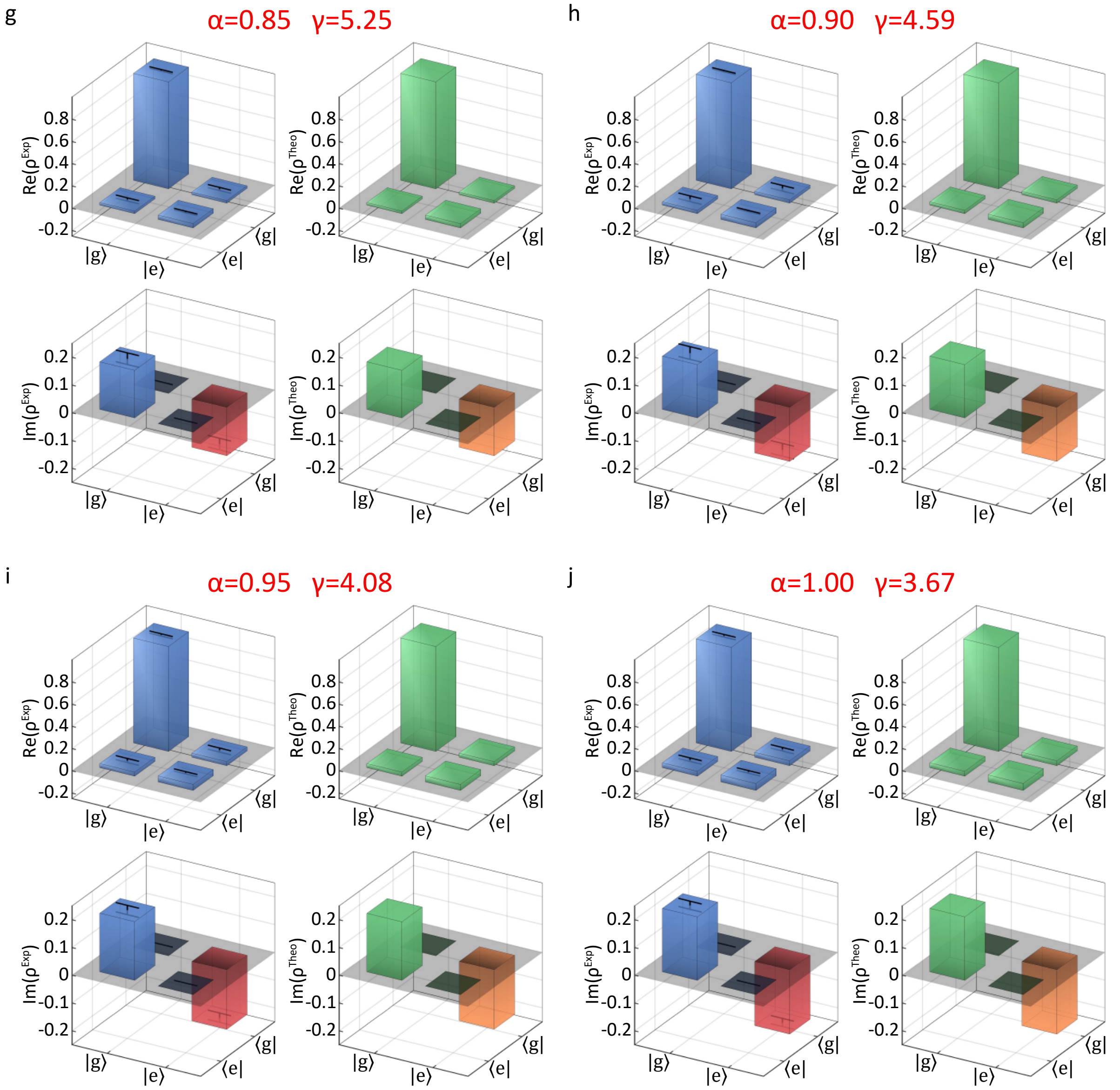

Supplementary Fig. 3. **Time evolution of the population as well as the steady-state tomography when $\Delta = \Omega/\sqrt{8}$.** (a) Time evolution of the population in $|e\rangle$ in z-direction with error bars. (b-j) The steady-state tomography with different α. The upper panels show the real parts while the lower panels display the imaginary parts. In each panel, the lefthand side are the experimental values with error bars and the righthand side are the theoretical values. Positive values are represented in blue(green) for experimental(theoretical) values, while negative values are shown in red(orange) to facilitate distinction. The error bars are standard deviation representing the statistical errors of 14 000 measurements for each data point.

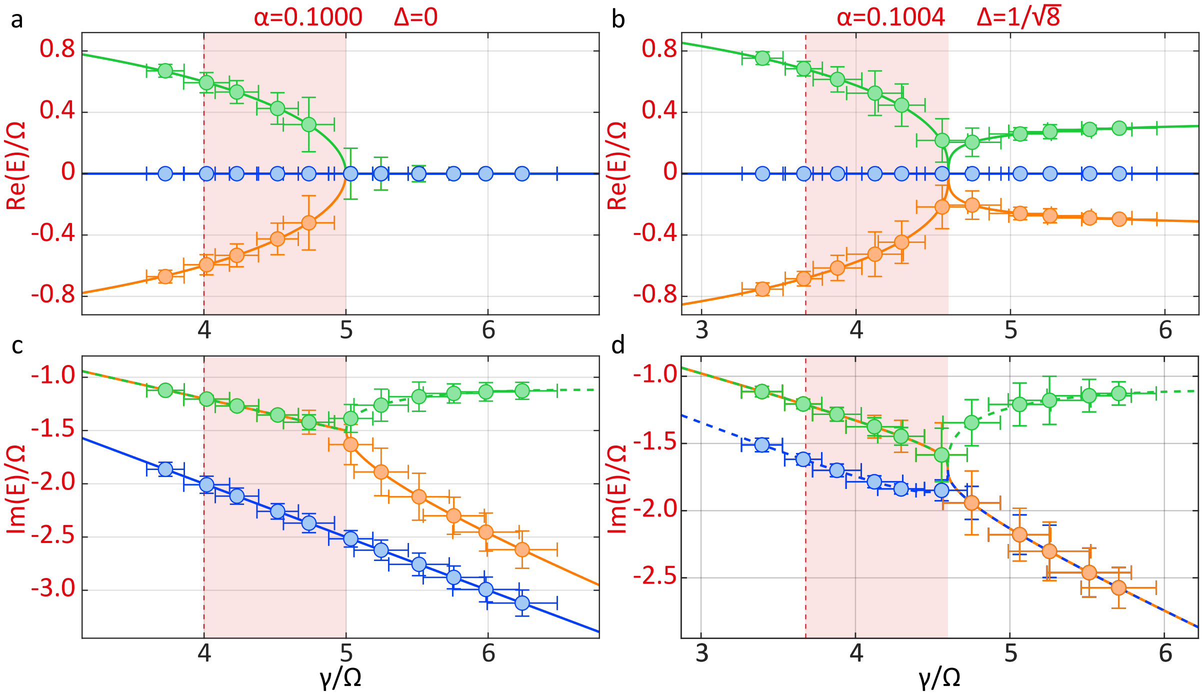

Supplementary Fig. 4. **Eigenenergies of second-order and third-order LEPs.** (a-d) The orange, blue and green dots with error bars are (a, b) the real and (c, d) the imaginary parts of the eigenvalues acquired from experimental measurement. The lines are calculated from the master equation. Red areas represent the movement range of the LEP with respect to the LEP at $\alpha = 0.1$. When $\Delta = 0$, the imaginary parts of the eigenenergies E_1 (orange line) and E_3 (green line) turn to be degenerate before the LEP is reached. When $\Delta = \Omega/\sqrt{8}$, E_1 (orange line) and E_3 (green line) become degenerate before reaching the LEP, while E_1 (orange line) and E_2 (blue line) become degenerate after crossing the LEP. For clarity, we replace some overlapped solid lines by dashed lines. The error bars represent standard deviation with the statistical errors of 14 000 measurements for each data point.

for $\Delta/\Omega = -1/\sqrt{8}$ as shown in Supplementary Fig. 5.

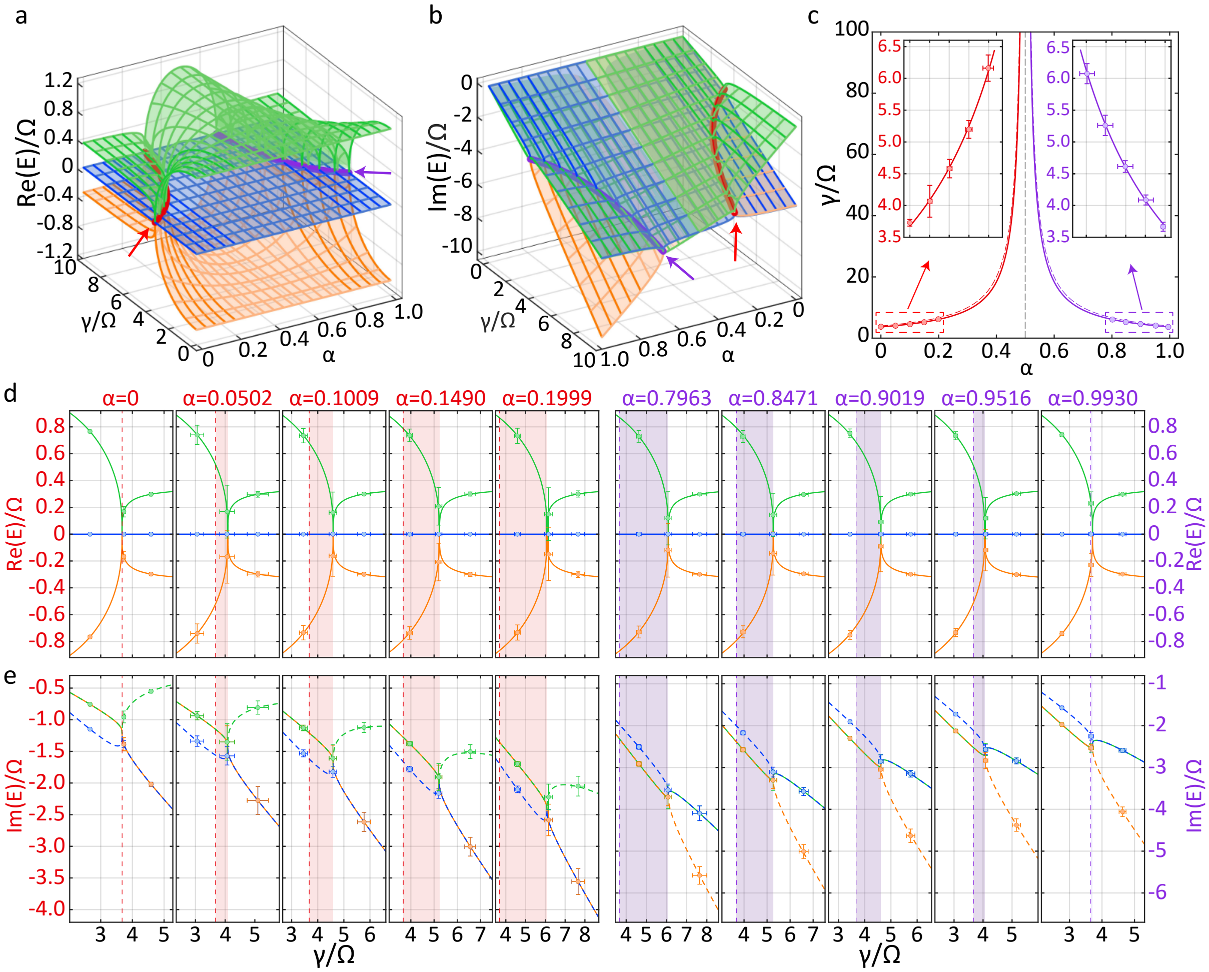

Supplementary Fig. 5. **Movement of third-order LEPs.** (a) The real part and (b) the imaginary part of the complex eigenenergy Riemann surfaces when $\Delta/\Omega = -1/\sqrt{8}$. The red and purple lines represent, respectively, the trajectories of the LEP's movement dominated by dephasing and decay. (c) The red and purple lines are theoretical calculation of the trajectories of the LEP's movement. Dots with error bars stand for the experimentally observed third-order LEPs with different α. In order to compare with the second-LEPs, we add red and purple dashed lines when $\Delta = 0$. (d-e) The orange, blue and green dots with error bars are (d) the real and (e) the imaginary parts of the eigenvalues acquired from experimental measurement. The lines are calculated from master equation. Red or purple area in each panel represents the movement range of the Lindblad LEP with α varying from $\alpha = 0$ (i.e., the dephasing LEP, labeled by a vertical dashed line) to the value labeled on the top of the panel. E_1 (orange) and E_3 (green) are degenerate before reaching LEP, while after crossing the LEP, E_1 (orange) and E_2 (blue) become degenerate when $\alpha < 0.5$, and E_2 (blue) and E_3 (green) become degenerate when $\alpha > 0.5$. For clarity, we replace some overlapped solid lines by dashed lines. The error bars represent standard deviation, i.e., the statistical errors of 14 000 measurements for each data point.

III. DERIVATIVES OF EIGENENERGIES $d(\mathrm{Re}[E])/d\alpha$ WITH RESPECT TO α

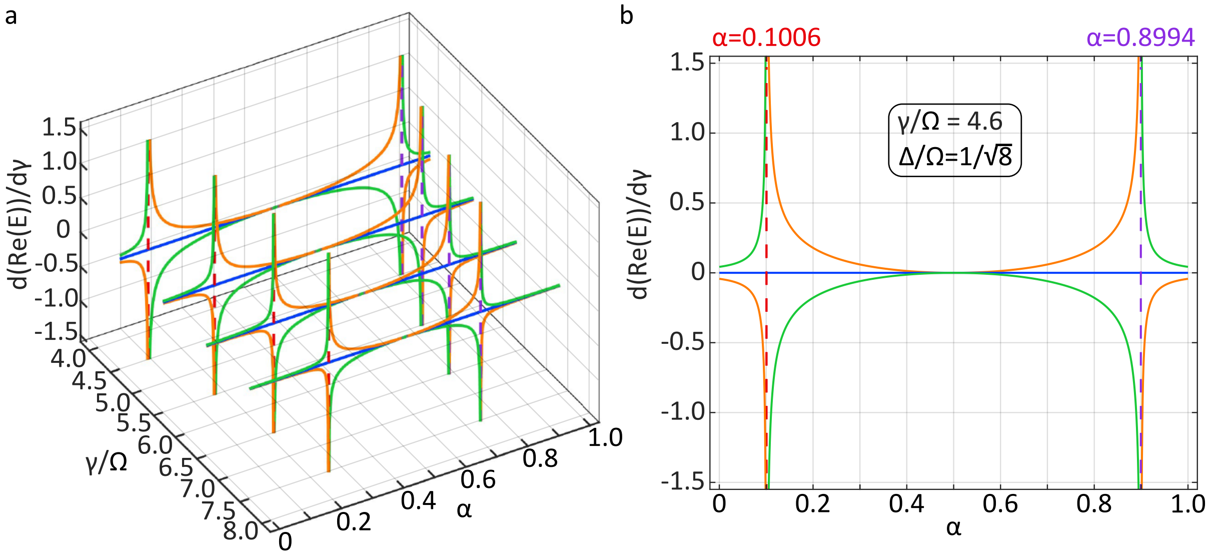

Supplementary Fig. 6. **Derivatives of the real parts of the eigenvalues $d(\mathrm{Re}[E])/d\alpha$ vs α.** (a) Derivatives of eigenenergies $d(\mathrm{Re}[E])/d\alpha$ as functions of α for different values of γ/Ω. (b) Two-dimensional map of panel (a), providing a clearer visualization of the derivatives of the real parts of the eigenvalues $d(\mathrm{Re}[E])/d\alpha$ vs α at fixed parameters $\gamma/\Omega = 4.6$ and $\Delta/\Omega = 1/\sqrt{8}$. The third-order LEPs are marked by vertical red and purple dashed lines, respectively, representing the Lindblad LEPs dominated by dephasing and decay. Here, the green, blue, and orange curves follow the same color coding as the eigenenergies $\mathrm{Re}[E]$ (from high to low) shown in Fig. 3d of the main text.

To have a straightforward demonstration of the derivatives of eigenenergies $d(\mathrm{Re}[E])/d\alpha$ with respect to α, we plot Supplementary Fig. 6 for the case of third-order LEPs. From these panels, we see more clearly that, as we approach the third-order LEPs, the slopes of the real part of the eigenvalue derivatives become steeper, indicating a stronger response to the signal being detected (see Supplementary Fig. 6a). For the system operating at $\gamma/\Omega = 4.6$, the positions of the third-order LEPs are given by $\alpha = 0.1006$ and $\alpha = 0.8994$ (see Supplementary Fig. 6b), according to Eq. (S4). Considering the experimental imperfections as demonstrated in Supplementary Fig. 3 of the main text, when $\gamma/\Omega = 4.6$, we deem that $\alpha = 0.1009$ and $\alpha = 0.9019$ are the optimal points in our experiment to exhibit the best sensitivity to the parameters being detected.
